# Rectal Cancer: Are 12 Lymph Nodes the Limit?

**DOI:** 10.3390/cancers15133447

**Published:** 2023-06-30

**Authors:** Paweł Mroczkowski, Łukasz Dziki, Tereza Vosikova, Ronny Otto, Anna Merecz-Sadowska, Radosław Zajdel, Karolina Zajdel, Hans Lippert, Olof Jannasch

**Affiliations:** 1 Department for General and Colorectal Surgery, Medical University of Lodz, Pl. Hallera 1, 90-647 Lodz, Poland; pawel.mroczkowski@umed.lodz.pl (P.M.); lukasz.dziki@umed.lodz.pl (Ł.D.); 2 Institute for Quality Assurance in Operative Medicine Ltd., Otto-von-Guericke-University, Leipziger Str. 44, D-39120 Magdeburg, Germany; tereza_vosikova@web.de (T.V.); ronny.otto@med.ovgu.de (R.O.); hans.lippert@med.ovgu.de (H.L.); olof.jannasch@gmx.de (O.J.); 3 Department for Surgery, University Hospital Knappschaftskrankenhaus, Ruhr-University, In der Schornau 23-25, D-44892 Bochum, Germany; 4 Department of Economic and Medical Informatics, University of Lodz, 90-214 Lodz, Poland; radoslaw.zajdel@uni.lodz.pl; 5 Department of Medical Informatics and Statistics, Medical University of Lodz, 90-645 Lodz, Poland; karolina.smigiel@umed.lodz.pl; 6 Department for General, Visceral and Vascular Surgery, Otto-von-Guericke-University, Leipziger Str. 44, D-39120 Magdeburg, Germany

**Keywords:** rectal cancer, lymph nodes, low anterior resection, abdominoperineal amputation

## Abstract

**Simple Summary:**

The study looked at the number of lymph nodes removed during rectal cancer surgery and whether the commonly recommended minimum of 12 nodes is necessary. The researchers analyzed data from 20,966 patients and found that factors such as age, gender and pre-therapeutic stage can affect the number of lymph nodes removed. The study also found that the probability of finding a positive lymph node increased with the number of nodes examined, suggesting that optimal surgical technique and pathological evaluation are more important than a numeric cut-off value.

**Abstract:**

Lymph node dissection is a crucial element of oncologic rectal surgery. Many guidelines regard the removal of at least 12 lymph nodes as the quality criterion in rectal cancer. However, this recommendation remains controversial. This study examines the factors influencing the lymph node yield and the validity of the 12-lymph node limit. Patients with rectal cancer who underwent low anterior resection or abdominoperineal amputation between 2000 and 2010 were analyzed. In total, 20,966 patients from 381 hospitals were included. Less than 12 lymph nodes were found in 20.53% of men and 19.31% of women (*p* = 0.03). The number of lymph nodes yielded increased significantly from 2000, 2005 and 2010 within the quality assurance program for all procedures. The univariate analysis indicated a significant (*p* < 0.001) correlation between lymph node yield and gender, age, pre-therapeutic T-stage, risk factors and neoadjuvant therapy. The multivariate analyses found T3 stage, female sex, the presence of at least one risk factor and neoadjuvant therapy to have a significant influence on yield. The probability of finding a positive lymph node was proportional to the number of examined nodes with no plateau. There is a proportional relationship between the number of examined lymph nodes and the probability of finding an infiltrated node. Optimal surgical technique and pathological evaluation of the specimen cannot be replaced by a numeric cut-off value.

## 1. Introduction

Rectal cancers are the second most prevalent tumors in the large intestine, following proximal colon cancers [1]. Consequently, rectal cancers have been regarded as a component of colorectal cancers (CRCs) in relevant epidemiological investigations. CRC ranks as the third leading cause of cancer-related mortality on a global scale and is the third most frequently diagnosed cancer [2]. Notably, a substantial decrease in the incidence of rectal cancer has been observed among individuals aged 65 and older. The overall incidence rate of rectal cancer is 11, whereas it amounts to 37.9 for patients under the age of 65. The overall incidence rate of rectal cancer is 13.9 in males and 8.6 in females, according to the data from 2015 to 2019 [1]. Rectal cancer incidence rates show a similar regional distribution, with particularly high rates observed in Eastern Asia. On the other hand, the incidence rates of rectal cancer tend to be low in most regions of Africa and South Central Asia. The overall 5-year survival rate for rectal cancer (66.5%) slightly surpasses that of colon cancer (64.2%), but stage-specific survival rates are comparable. There is no significant variation in survival rates based on gender. The largest sex disparity in 5-year survival is for left-sided colon cancer, at 67% in men versus 70% in women [1,3].

Numerous investigations and comprehensive analyses have extensively addressed the spectrum of risk factors associated with CRCs. Nonetheless, only a restricted subset of these studies has endeavored to disentangle the distinctive contributions of environmental and genetic factors, which possess the potential to influence the predisposition towards colon and rectal cancers. It is crucial to acknowledge that age and gender represent pivotal risk determinants that exert their influence on both colon and rectal cancers. Furthermore, empirical evidence has substantiated that a hereditary lineage characterized by a history of colorectal cancer notably impacts the risk of developing colon cancer to a greater extent as opposed to rectal cancer [4]. However, the modulation of rectal cancer risk may occur through the involvement of gene polymorphisms. Makar et al. demonstrated that the rs20417 polymorphism in the cyclooxygenase 2 (*COX-2*) gene is associated with a higher risk of rectal cancer [5]. Conversely, Liu et al. showed that polymorphism rs24384 in the matrix metallopeptidase 2 (*MMP2*) gene is associated with a decreased risk of rectal cancer [6]. Similarly, other polymorphisms that are related to decreased risk of rectal cancer are in the methylenetetrahydrofolate reductase (*MTHFR*) gene (rs1801133) [7] and the peroxisome proliferators-activated receptor gamma (*PPARγ*) gene (rs1801282) [8]. Additionally, environmental influences, including dietary patterns and physical activity levels, are recognized as crucial factors that can also modulate the likelihood of developing rectal cancer [9].

While the current screening programs implemented worldwide have successfully identified a considerable proportion of asymptomatic cases in the early stages, it is noteworthy that a substantial number of diagnoses occur subsequent to the manifestation of symptoms. Among these symptoms, rectal bleeding emerges as the prevailing presentation associated with rectal cancer. As the disease progresses to later stages, additional manifestations such as tenesmus, incomplete stool evacuation, reduced stool caliber, cramping, pelvic and rectal pain, as well as obstructive symptoms, may become evident. Upon careful examination of the presenting symptoms pertaining to CRCs as a whole, it becomes apparent that the clinical manifestations vary contingent upon the precise location of the tumor [3,10]. In scientific literature, the pathological stage holds paramount significance in predicting the prognosis of individuals diagnosed with rectal cancer. The widely adopted staging system for this purpose is the tumor-node-metastasis (TNM) system, established by the esteemed American Joint Committee on Cancer (AJCC). This system primarily takes into account the depth of local invasion, the extent of regional lymph node engagement, and the presence of distant sites of the disease [11]. Notably, as the AJCC stage progresses from stage I to stage IV, the 5-year overall survival rate experiences a substantial decline, plummeting from over 90% to below 10% [12].

Surgical intervention aimed at achieving a curative outcome presents the most favorable prospects for prolonged survival in cases of rectal adenocarcinoma. The anatomical constraints imposed by the bony pelvis have necessitated the development of various innovative surgical techniques for rectal cancer, marking significant milestones in the field. These advancements have contributed to notable enhancements in local recurrence rates and a simultaneous reduction in the overall burden of morbidity and mortality. The treatment of rectal cancer is based on perioperative radiotherapy and chemotherapy with standard management in operable tumors, i.e., surgical resection followed by lymph node (LN) dissection. As such, LN dissection is a crucial element of oncologic surgery [13,14].

Neoadjuvant therapy combines radiotherapy and chemotherapy as a treatment approach. The European Society of Medical Oncology (ESMO) recommends neoadjuvant therapy for cases of advanced disease (>cT3), lymph node involvement observed on imaging, and situations where the adequacy of TME (total mesorectal excision) surgery is uncertain, particularly in relation to the circumferential resection margin. The primary objective of neoadjuvant therapy is to reduce the size or stage of the tumor prior to surgical removal. In some instances, tumors may exhibit a complete response to neoadjuvant therapy, characterized by the replacement of the tumor with fibrous tissue following radiotherapy. The decision to administer neoadjuvant therapy to a patient depends on the clinical stage of the tumor at the time of diagnosis [15,16].

The selection and scope of surgical procedures conducted on individuals with rectal cancer are predominantly determined by several factors, including the preoperative stage of the tumor, the proximity to the anorectal sphincter complex, the utilization of neoadjuvant therapy, histopathological characteristics, and the patient’s anticipated capacity to withstand extensive surgical intervention. The technique of total mesorectal excision (TME) performed in concert with abdominoperineal resection (APR) or low anterior resection (LAR) allows for precise dissection and removal of the entire rectal mesentery, including that distal to the tumor, as an intact unit. Dissection in the mesorectal plane must include blood vessels, lymphatic vessels and lymph nodes through which the tumor can spread [17,18,19]. TME techniques have reduced recurrence rates to 6–12% and extended 5-year survival to 53–87%, according to various literature data [20,21,22,23]. The LNs are distributed above (53%), adjacent (36%) or below (11%) the tumor and their diameter may not exceed 3 mm [24].

A crucial prognostic factor for long-term outcome is the presence of LN metastases [25,26]. According to the AJCC, patients characterized by a positive number of LNs (N staging) can be divided into two groups: those with 1–6 LN metastases are included in group N1, and those with seven or more are in group N2. Moreover, the automated linear model proposes that the number of positive LNs is related to tumor size and differentiation, tumor invasion, chemotherapy and TNM staging [27].

The implementation of a more aggressive or extensive lymphadenectomy approach for rectal cancer has been suggested as a potential strategy for enhancing local disease control and overall outcomes [28]. Nevertheless, while there is evidence in favor of extended lymphadenectomy in rectal cancer [29], another study has indicated that this approach does not yield a statistically significant advantage in terms of survival or recurrence rates for patients with locally advanced primary or recurrent rectal cancer [30]. Recent guidelines suggest removing at least 12 lymph nodes during surgery for rectal cancer [31]; however, this strategy remains controversial [32,33,34,35]. Furthermore, in the case of neoadjuvant treatment, the number of yielded lymph nodes may often be reduced [33,34,36,37]. There is clearly a need for greater clarity regarding this issue. Therefore, the present study examines the factors influencing lymph node yield and the validity of the 12-lymph node recommendation.

## 2. Materials and Methods

The cohort included patients with rectal cancer recorded in the international quality assessment project for colon cancer in Germany. All had undergone low anterior resection (LAR) or abdominoperineal amputation (APR) between 1 January 2000 and 31 December 2010. The concept of the project was described previously [38]. The enrollment questionnaire encompassed various aspects of patient information, including risk factors, reasons for hospitalization, preoperative diagnostics, surgical procedures, intraoperative complications, general and surgical postoperative complications, pathological reports and discharge status.

Included were all patients with histopathologically verified rectal cancer. Histological examinations were conducted by the local pathologist; these included the tumor stage and the number of detected and infiltrated lymph nodes. Exclusion criteria were anal cancer, tumors localized more than 16 cm from the anal verge, treatment outside of Germany, unknown lymph node status and TNM stage IV.

Patient sex, age, body mass index, mean stay in hospital, morbidity and mortality were recorded, as well as tumor TNM stage. In addition, a number of therapy-related factors were recorded: neoadjuvant treatment, intraoperative complications (tumor perforation, bleeding, lesions of the urethra, ureter, urinary bladder, spleen, intestine, interior genitals and complications of the anastomosis), and general risk factors.

To evaluate the impact of participation in the quality assessment project, the results for the years 2000, 2005 and 2010 were compared separately.

Descriptive statistics were calculated: absolute incidences for categorical variables, arithmetic mean and standard deviation, as well as median values. The Chi-square test was used to verify the association between two categorical variables. For continuous variables, data that were normally distributed were compared using a t-test, while data that was not was subjected to the Mann–Whitney U-test. Linear correlation between the probability of finding an infiltrated lymph node and the number of analyzed lymph nodes was determined using Pearson’s correlation coefficient. All parameters were subjected to univariate analysis. Any significant parameters then underwent multivariate analysis. In addition, odds ratios with 95% confidence intervals were calculated for each risk factor. A *p*-value of <0.05 was considered statistically significant.

The statistical analysis was performed with IBM^®^ SPSS^®^ Statistics, Version 21.0.0; SPSS Inc. (New York, NY, USA).

## 3. Results

In total, 20,966 patients from 381 hospitals fulfilled the inclusion criteria. The group contains 12,446 men and 8,520 women, with a mean age of 66.85 ± 10.45 years. Less than 12 lymph nodes were yielded in 20.53% of the men and 19.31% of the women (*p* = 0.03). LNs < 12 group was also older (67.2 ± 10.2 years vs. 66.5 ± 10.7 years; *p* < 0.001). Detailed characteristics of the patients, divided into LNs < 12 and LNs ≥ 12 groups, are given in Table 1.

The number of yielded lymph nodes for all procedures increased significantly between the years 2000, 2005 and 2010. In 2010, this median number amounted to 18.57 for all procedures. The results are summarized in Table 2.

The univariate analysis discovered a significant (*p* < 0.001) correlation between the lymph node yield and patient sex, age, pre-therapeutic T-stage (cT), risk factors and neoadjuvant therapy. There is no significant correlation between the lymph node yield, patient BMI and intraoperative complications. The detailed analysis is presented in Table 3.

In the multivariate analyses, pre-therapeutic cT-stage, female sex, the presence of at least one risk factor and neoadjuvant therapy had a significant influence on the yield (Table 4).

**Figure 1:**
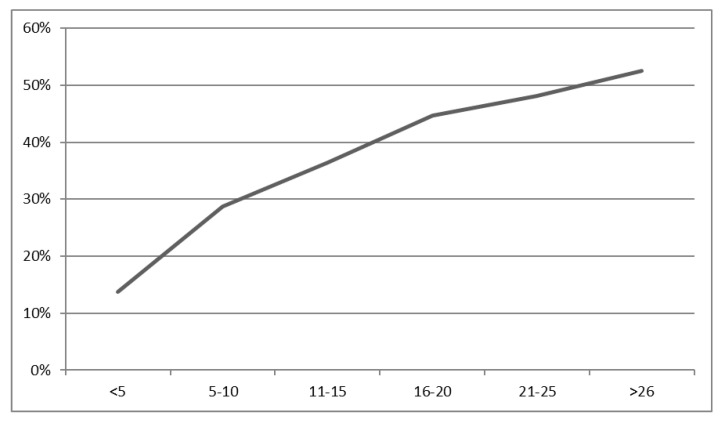
Relationship between the probability of finding an infiltrated lymph node and the number of analyzed lymph nodes in percent. *p* for the Chi-square test < 0.001.

The probability of finding a positive lymph node was proportional to the number of examined nodes. At least one positive node was found in 13.7% of the specimens with five or fewer investigated lymph nodes and in 52.5% of the specimens with 25 or more (*p* < 0.001). No plateau was reached. More detailed results are shown in Figure 1.

## 4. Discussion

Throughout the years, numerous studies utilizing retrospective cohort data and administrative claims data have presented evidence of enhanced survival rates among patients with rectal cancer who underwent examination of a greater number of nodes following resection. Several observational studies have identified a correlation between the assessment of a “sufficiently” deemed number of lymph nodes and improved survival outcomes. Notably, this therapeutic advantage has been suggested specifically for individuals with confirmed lymph node involvement. However, it is important to note that the relationship between lymph node counts and survival in rectal cancer does not consistently demonstrate uniformity.

In the surgical management of rectal cancer, it is essential to achieve a clear circumferential resection margin and distal resection margin. The objective of the study conducted by Zedan and Salah was to assess the morbidity, mortality, survival outcomes and local failure rates following TME in the surgical treatment of rectal cancer. This retrospective analysis included 101 patients who underwent LAR, APR, or Hartmann′s technique for rectal cancer. TME was performed in all cases. Of the 101 evaluable patients, 61 were males and 40 were females. The distribution of operative procedures was as follows: APR in 15.8% of patients, LAR in 71.3% of patients, and Hartmann′s technique in 12.9% of patients. The 30-day postoperative mortality rate was 3%. Overall, 25% of patients experienced postoperative morbidity, including an anastomotic site leak in 5.9% of patients, urinary dysfunction in 9.9% of patients, and erectile dysfunction in 15.8% of male patients. The median distances for the safety margin were 23 mm for the distal margin and 12 mm for the radial margin, with a median distal limit of 7 cm. The median number of harvested lymph nodes was 19. Tumor locations were as follows: anteriorly in 23.8% of cases, laterally in 13.9% of cases, posteriorly in 38.6% of cases, and circumferentially in 23.8% of cases. Regarding the TNM classification, 3% had T1 tumors, 28.7% had T2 tumors, 55.4% had T3 tumors, and 12.9% had T4 tumors. Nodal involvement was present in 57.4% of cases (N1 in 31.7% and N2 in 10.9%). TNM staging revealed that 15.8% were classified as stage I, 29.7% as stage II, 46.5% as stage III, and 7.9% as stage IV. Chemotherapy was administered to 67.3% of patients, while radiotherapy (short-course neoadjuvant, long-course neoadjuvant and adjuvant postoperative) was used in 33.7%, 20.8% and 19.8% of patients, respectively. The 5-year cancer-specific survival rate was 73%, and the 5-year recurrence-free survival rate was 71%. Therefore, TME is considered the gold standard technique in rectal cancer surgery. It ensures safety when combined with neoadjuvant chemoradiotherapy and offers optimal oncological outcomes, including local control, long-term survival and preservation of a good quality of life [39].

The evaluation of lymph node engagement in individuals diagnosed with rectal cancer plays a crucial role in the management of the disease [40]. Among patients with T1 tumors, the presence of positive lymph nodes was observed in 12.2% of cases, while for patients with T2 tumors, the corresponding percentage was 18.0% [41]. Previous univariate analyses indicate that tumor size, grading, stage, number of harvested lymph nodes, tumor aggregation within vessels, and peripheral nerve infiltration are LN metastasis risk factors [42]. The results indicate that metastatic LNs generally occur in all size categories [43]. The findings also indicate that the presence of lymphatic vessels in regional lymph nodes has a significant impact on disease-free survival. However, no correlation was found between peritumoral or intratumoral lymph vessel density and prognosis in rectal cancer patients who were treated with neoadjuvant radiochemotherapy and consecutive curative surgery [44].

Many guidelines set the removal of at least 12 lymph nodes as a cut-off value to ensure accurate staging and adequate therapy of colorectal cancer [31,45,46]. Wang et al. demonstrated that in patients with locally advanced rectal cancer who received neoadjuvant chemoradiotherapy, a lymph node yield of at least 12 was associated with improved survival. However, a lower lymph node yield did not correlate with enhanced tumor regression. These findings suggest that a sufficiently high lymph node yield is still necessary, particularly in individuals who may have a poor tumor response [47].

The probability of finding at least one infiltrated lymph node was found to increase with the number of nodes yielded. This observation confirms those of Lykke et al. [32]. A median lymph node yield of 10 and 15 and rates of node-positive disease of 31.6% and 36.7% were observed with and without neoadjuvant treatment, respectively. However, Govindarajan et al. report lower probabilities, i.e., 10.88% in a group with zero to three lymph nodes and 31.4% among those with more than twenty lymph nodes, but like our results, no plateau was observed [48]. Persiani et al. could not identify any significant correlation between the number of retrieved lymph nodes and the rate of node positivity; however, patients with a yield of 12 or more nodes yielded higher numbers of positive lymph nodes [49].

Our data indicate that the tested women had a higher probability (RR 1.13) of reaching a lymph node yield of 12. This result is similar to those of Ahmadi et al. and Govindarajan et al. [48,50]; however, several studies indicate no correlation between these factors [51,52,53]. Others report an increase in lymph node yield with decreasing age [50,51,52,54], whereas Amajoyi et al., Scabini et al. and Persiani et al. found no such correlation [49,55,56]. Also, in our cohort, neither age nor body mass index were found to be significant in the multivariate analysis; this finding correlates with previous findings [52,53,54].

In the present study, the absence of general risk factors and higher cT-stages were associated with a higher probability of yielding at least 12 nodes. The positive correlation between cT-stage and lymph node yield has also been described by other authors [33,57,58]. Our present findings also indicate the greatest difference for the T3 stage (T2 RR 1.800, T3 RR 2.888, and T4 RR 2.172), as confirmed by Chou et al. [59].

The only therapy-dependent significant factor was neoadjuvant therapy—patients that did not receive a neoadjuvant treatment had a 2.7 times higher probability of a yield of at least 12 lymph nodes. Previous linear regression analyses of neoadjuvant therapy on lymph node yield have yielded coefficients of −5.937 [60] and −5.56 [55]. Other authors also described a lower lymph node yield after neoadjuvant radiochemotherapy [48,61,62,63].

Our study did not reveal a cut-off value for the number of examined lymph nodes. However, a number of cut-off values have been proposed in the literature, ranging from eight lymph nodes [34] to ten lymph nodes [64], four to fifteen lymph nodes [35] or even twenty lymph nodes [65]; however, several authors have confirmed the suggestion of twelve lymph nodes [18,66]. Inadequate LN harvest was noted in 43.8% of patients before 2008 when guidelines for the removal of 12 lymph nodes emerged; however, this rate declined to 18.4% in later years [67].

Our findings confirm previous observations that the year of treatment seems to have a considerable influence on the lymph node yield [35,51,55]. The mean number of yielded lymph nodes increased significantly over time, from 16.03 lymph nodes in 2000 up to 18.57 lymph nodes in 2010. Our results revealed higher lymph node yields than other studies, noting, for example, 12.1 lymph nodes [68], 12.8 lymph nodes [69], 14.6 lymph nodes [52], 14 lymph nodes [50] or 15 lymph nodes [36]. This increase in the number of nodes may reflect a heightened desire to improve surgical quality as part of the quality assurance project [70,71]. The number of analyzed nodes could be an indicator of the quality of surgery and pathological examination [72,73,74].

The main limitation of the study was the non-standardized handling of the pathological specimen. As the pathological evaluation was performed in local hospitals, no detailed information about the employed technique was collected. Different techniques have different potentials to detect lymph nodes [75].

Lymph node infiltration plays a crucial role in the staging and therapy of rectal cancer, and as such, correct identification of nodal positivity is essential. Analyzing twelve nodes may be insufficient as a surgical goal, and every effort should be employed to find and analyze all nodes in the specimen without any limitations imposed by predefined cut-off values. Every additional removed and analyzed lymph node increases the probability of finding an infiltrated one and of addressing the disease properly.

## 5. Conclusions

There is a proportional relationship between the number of examined lymph nodes and the probability of finding an infiltrated node. Optimal surgical technique and pathological evaluation of the specimen cannot be replaced by a numeric cut-off value.

## Figures and Tables

**Table 1:** Detailed patient characteristics.

	Total (2000–2010)		*p*
lymph node yield	*n* < 12	*n* ≥ 12	
patients *n* (%)	20,966		
male *n* (%)	2555 (20.53%)	9891 (79.47%)	0.030
female *n* (%)	1645 (19.31%)	6875 (80.69%)	
mean age ± SD (years)	67.2 ± 10.2	66.5 ± 10.7	<0.001
mean body mass index ± SD (kg/m^2^)	26.4 ± 4.2	26.3 ± 4.2	0.991
mean stay in hospital ± SD (days)	21.0 ± 11.7	21.5 ± 13.2	0.417
morbidity *n* (%)	1648 (39.24%)	6634 (39.57%)	0.696
mortality *n* (%)	95 (0.45%)	401 (1.91%)	0.621

*p* for the Chi-square test (gender, morbidity and mortality) and *p* for the Mann–Whitney U-test (age, body mass index and stay in hospital)

**Table 2:** Average lymph node yield in selected years.

	2000	2005	2010	*p*
APR	14.68	17.55	18.65	<0.001
LAR	16.57	17.51	18.55	<0.001
LAR with pouch	16.48	16.33	18.49	0.039
total	16.03	17.70	18.57	<0.001

*p* for the Chi-square test.

**Table 3:** Parameters for the yield of at least 12 lymph nodes—univariate analysis.

	*n*	<12 Lymph Nodes	≥12 Lymph Nodes	*p*
sex (*n*)				
male	12,446	2555 (20.5%)	9891 (79.5%)	0.030
female	8520	1645 (19.3%)	6875 (80.7%)	
BMI (kg/m^2^)				
<18.5	330	63 (19.2%)	267 (80.8%)	0.334
18.5–25	8159	1599 (19.6%)	6560 (80.4%)	
25.1–30	8925	1830 (20.5%)	7095 (79.5%)	
>30	3552	686 (19.3%)	2866 (80.7%)	
age (years)				
<63	6739	1247 (18.5%)	5492 (81.5%)	
63–71	7137	1492 (20.9%)	5645 (79.1%)	<0.001
>71	7090	1468 (20.7%)	5622 (79.3%)	
cT				
cT1	1549	431 (27.8%)	1118 (72.2%)	<0.001
cT2	6277	1205 (19.2%)	5072 (80.8%)	
cT3	12,333	2245 (18.2%)	10,088 (81.8%)	
cT4	807	178 (22.0%)	629 (78.0%)	
general risk factors				
no	4708	852 (18.1%)	3856 (81.9%)	<0.001
≥1	16,258	3349 (20.6%)	12,909 (79.4%)	
intraoperative complications				
no	19,827	3965 (20.0%)	15,862 (80.0%)	0.215
≥1	1139	245 (21.5%)	894 (78.5%)	
neoadjuvant radiochemotherapy				
no	16,569	2999 (18.1%)	13,570 (81.9%)	<0.001
yes	4397	1183 (26.9%)	3214 (73.1%)	

*p* for the Chi-square test

**Table 4:** Parameters for reaching a yield of at least 12 lymph nodes—multivariate analysis.

	*p*	Odds Ratio	95% Confidence Interval
cT1		1	
cT2	<0.001	1.800	1.493–2.171
cT3	<0.001	2.888	2.383–3.500
cT4	<0.001	2.172	1.594–3.032
female	0.020	1.135	1.020–1.262
no general risk factor	<0.001	1.390	1.227–1.574
no neoadjuvant	<0.001	2.685	2.379–3.032

*p* for the Chi-square test

## Data Availability

The data presented in this study are available in this article.
